# Identification of Inhibitory Ca^2+^ Binding Sites in the Upper Vestibule of the Yeast Vacuolar TRP Channel

**DOI:** 10.1016/j.isci.2018.11.037

**Published:** 2018-12-04

**Authors:** Mahnaz Amini, Hongmei Wang, Anouar Belkacemi, Martin Jung, Adam Bertl, Gabriel Schlenstedt, Veit Flockerzi, Andreas Beck

**Affiliations:** 1 Experimentelle und Klinische Pharmakologie und Toxikologie, PZMS, Universität des Saarlandes, 66421 Homburg, Germany; 2 Medizinische Biochemie und Molekularbiologie, PZMS, Universität des Saarlandes, 66421 Homburg, Germany; 3 Fachbereich Biologie, Technische Universität Darmstadt, 64287 Darmstadt, Germany

**Keywords:** Structural Biology, Protein Structure Aspects, Biophysics

## Abstract

By vacuolar patch-clamp and Ca^2+^ imaging experiments, we show that the yeast vacuolar transient receptor potential (TRPY) channel 1 is activated by cytosolic Ca^2+^ and inhibited by Ca^2+^ from the vacuolar lumen. The channel is cooperatively affected by vacuolar Ca^2+^ (Hill coefficient, 1.5), suggesting that it may accommodate a Ca^2+^ receptor that can bind two calcium ions. Alanine scanning of six negatively charged amino acid residues in the transmembrane S5 and S6 linker, facing the vacuolar lumen, revealed that two aspartate residues, 401 and 405, are essential for current inhibition and direct binding of ^45^Ca^2+^. Expressed in HEK-293 cells, a significant fraction of TRPY1, present in the plasma membrane, retained its Ca^2+^ sensitivity. Based on these data and on homology with TRPV channels, we conclude that D401 and D405 are key residues within the vacuolar vestibule of the TRPY1 pore that decrease cation access or permeation after Ca^2+^ binding.

## Highlights

• The yeast vacuolar TRPY1 channel is inhibited by vacuolar Ca^2+^
• Aspartate residues D401A and D405A are essential for Ca^2+^-mediated inhibition
• Aspartate residues D401 and D405 are essential for direct Ca^2+^ binding
• Ca^2+^ binding to D401 and D405 within vacuolar pore vestibule mediates inhibition

## Graphical Abstract

**Figure:**
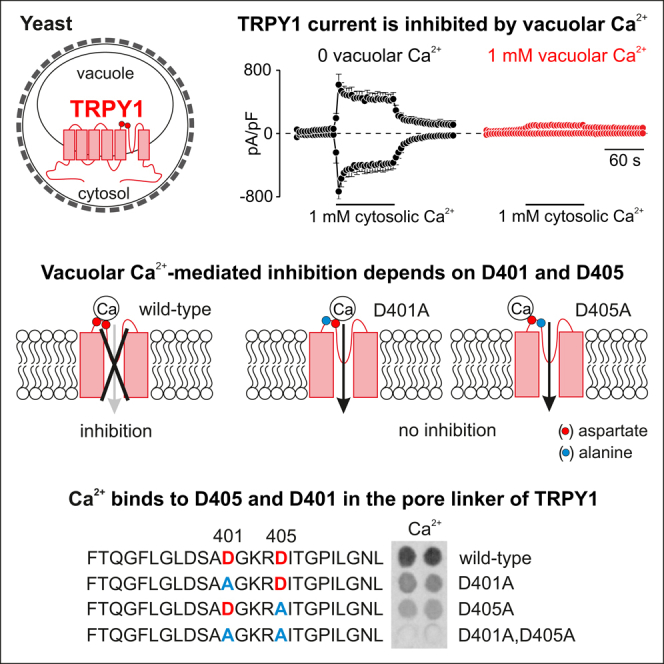

## Introduction

The transient receptor potential (TRP) ion channel family is encoded by more than 100 genes (Venkatachalam and Montell, 2007). With the exception of the Ca^2+^-selective TRPV5 and TRPV6 the TRP channels are non-selective cation channels sharing the membrane topology of six transmembrane helices (S1–S6) and N- and C-terminal domains residing within the cytosol. By mediating cation influx, TRP channels shape the membrane potential and increase the cytosolic Ca^2+^ concentration ([Ca^2+^]_cyt_), translating environmental and endogenous stimuli into cellular signals. Most TRP channels fulfill their physiological function in the plasma membrane as cation influx channels, whereas some functional TRP channels are localized in the membrane of cytoplasmic organelles (Berbey et al., 2009, Chen et al., 2014, Dong et al., 2008, Lange et al., 2009, Oancea et al., 2009, Turner et al., 2003).

Calcium ions permeate TRP channels - with the exception of the Ca^2+^-impermeable TRPM4 and TRPM5 - and also potentiate, activate, or inhibit currents most probably by interfering with TRP channel domains facing the cytosol (Blair et al., 2009, Bodding et al., 2003, Doerner et al., 2007, Du et al., 2009, Gross et al., 2009, Launay et al., 2002, McHugh et al., 2003, Olah et al., 2009, Prawitt et al., 2003, Starkus et al., 2007, Voets et al., 2002, Watanabe et al., 2003, Xiao et al., 2008, Zurborg et al., 2007). Likewise the TRP channel TRPY1, encoded by the vacuolar conductance 1 (*YVC1*) gene in *Saccharomyces cerevisiae* and localized in the vacuolar membrane, is activated by Ca^2+^ interfering with cytosolic channel domains (Bertl and Slayman, 1990, Palmer et al., 2001, Wada et al., 1987). TRPY1 mediates vacuolar Ca^2+^ release, and the amount of cytosolic [Ca^2+^] required for TRPY1 activation can be substantially lowered in the presence of reducing agents such as dithiothreitol (DTT), glutathione, or β-mercaptoethanol (Bertl et al., 1992b, Bertl and Slayman, 1990). In addition to Ca^2+^, TRPY1 is also permeable to monovalent cations (permeability ratio P_Ca_/P_K_ ∼5; Bertl and Slayman, 1992), revealing a single-channel conductance of more than 300 pS in 180 mM KCl (Chang et al., 2010, Palmer et al., 2001, Wada et al., 1987, Zhou et al., 2003). TRPY1 is suggested to be involved in the response to hyperosmotic and oxidative stress as well as glucose-induced Ca^2+^ signaling (Bertl and Slayman, 1990, Bouillet et al., 2012, Denis and Cyert, 2002, Palmer et al., 2001). However, the exact function of the TRPY1-mediated Ca^2+^ release is still elusive.

Binding of Ca^2+^ by some mammalian TRP channels including the Ca^2+^-activated TRPM4 has been described to be mediated by four coordinating residues within S2 and S3 close to the cytosolic S2-S3 linker (Autzen et al., 2018). These residues are not conserved in the TRPY1 sequence. All mammalian TRPs and the fly's TRPL are binding Ca^2+^/calmodulin *in vitro,* and Bertl et al. (Bertl et al., 1992b, Bertl et al., 1998a, Bertl et al., 1998b) suggested that Ca^2+^/calmodulin contributes to TRPY1 activation in yeast, but a negative charge cluster, D^573^DDD^576^, within the TRPY1's cytosolic C-terminus was shown to be crucial for the Ca^2+^-mediated activation of TRPY1 (Su et al., 2009).

In the present study, we characterized the dependence of TRPY1 current inhibition and activation on vacuolar and cytosolic [Ca^2+^] by patch-clamp recordings from yeast vacuoles and Ca^2+^ imaging in yeast. By alanine scanning and direct ^45^Ca^2+^ binding, we identified two aspartate residues within the S5-S6 linker facing the vacuolar lumen to be essential for Ca^2+^ binding and Ca^2+^-dependent inhibition of the TRPY1 current.

## Results

### Cytosolic Ca^2+^ Activates the Vacuolar TRPY1 Channel

**Figure 1:**
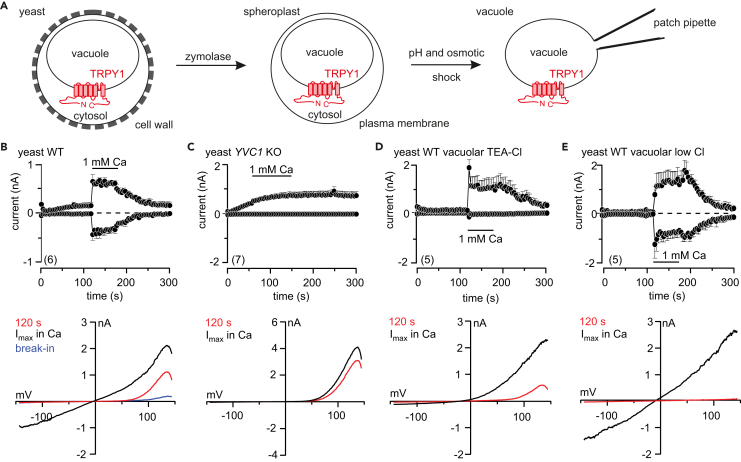
Cytosolic Ca^2+^ Activates Cation Conductance in Yeast Vacuoles via TRPY1 (A) Procedure to release the yeast vacuole for patch-clamp experiments. (B–E) Inward currents at −80 mV and outward currents at 80 mV, extracted from 200-ms ramps (0.5 Hz) spanning from 150 to −150 mV, V_h_ = 0 mV, plotted versus time (top), and corresponding current-voltage relations (IVs) at break-in (blue), after 120 s (red) and at maximum current (I_max_) in 1 mM cytosolic Ca^2+^ (black; bottom) measured in isolated vacuoles from wild-type (WT; B, D, and E) and *YVC1*-deficient (*YVC1* KO; C) yeast. The membrane potentials refer to the cytosolic side, i.e., inward currents represent movement of positive charges from the vacuole toward the cytosol (Bertl et al., 1992a). 1 mM Ca^2+^ was applied to the bath (representing the cytosol) as indicated by the bars. In (D) K^+^ and in (E) Cl^−^ were substituted by TEA^+^ (tetraethylammonium) and gluconate in the patch pipette, respectively. Currents and IVs are shown as means ± SEM and just means, respectively, with the number of measured cells indicated in brackets.

**Figure 2:**
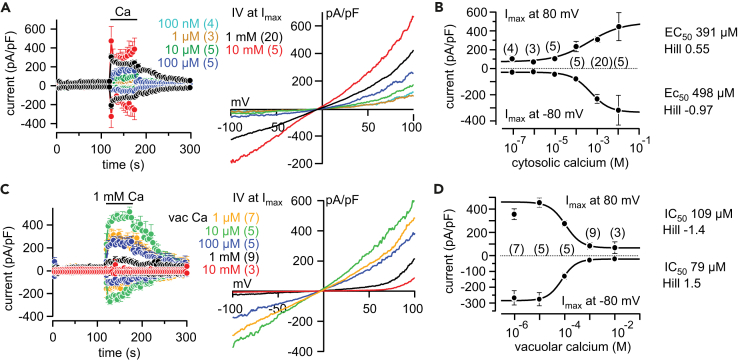
Concentration-Dependent Activation of Yeast Vacuolar TRPY1 by Cytosolic Ca^2+^ and Inhibition by Vacuolar Ca^2+^ (A–D) Inward and outward currents at −80 and 80 mV, respectively, extracted from 200-ms ramps (0.5 Hz) spanning from 150 to −150 mV, V_h_ = 0 mV, plotted versus time (A and C, left), and corresponding IVs at maximum currents (I_max_, A and C, right) activated by diverse cytosolic Ca^2+^ concentrations ([Ca^2+^]_cyt_, A and B) and 1 mM [Ca^2+^]_cyt_ at the indicated vacuolar Ca^2+^ concentrations ([Ca^2+^]_vac_, vac Ca, corresponding to the [Ca^2+^] in the patch pipette, C and D) in isolated vacuoles from wild-type yeast. Application of Ca^2+^ is indicated by the bar. In (B), maximal inward currents (at −80 mV) and outward currents (at 80 mV) are plotted versus the [Ca^2+^]_cyt_ concentration. The sigmoidal fits reveal EC_50_ values of 498 and 391 μM [Ca^2+^]_cyt_ for half-maximal activation of the inward and outward currents (B), respectively. In (D), currents at −80 and 80 mV activated by 1 mM [Ca^2+^]_cyt_ are plotted versus the [Ca^2+^]_vac_. The sigmoidal fits reveal half-maximal inhibition by [Ca^2+^]_vac_ of 79 and 109 μM for the inward and outward currents (D), respectively. Currents are normalized to the size of the vacuole (pA/pF) and shown as means ± SEM (A–D) and means (IVs in A and C), with the number of measured cells indicated in brackets. See also Figures S1 and S2.

To get electrophysiological access to the yeast vacuole, the cell wall and membrane have to be removed (Figure 1A). At break-in and application of voltage ramps (150 to −150 mV), a small voltage-dependent outward rectifying current was recorded at membrane potentials above 80 mV (Figure 1B, bottom, blue current-voltage relation [IV]). Membrane potentials refer to the cytosolic side, and inward currents are defined as cation flow across the vacuolar membrane into the cytosol (Bertl et al., 1992a). Upon perfusion of the 150 mM KCl pipette solution, i.e., washout of vacuolar content, the outward current increases to maximum amplitude within 120 s (Figure 1B, red IV). Application of 1 mM cytosolic Ca^2+^ ([Ca^2+^]_cyt_) activates additional inward and outward currents (Figure 1B, I_max_ in Ca), which are absent in vacuoles isolated from *YVC1* knockout cells (*YVC1* KO; Figure 1C). These currents are carried by cations, as their inward portion disappears in the absence of vacuolar (pipette) K^+^ substituted by TEA^+^ (tetraethylammonium) (Figure 1D). In contrast, the voltage-dependent outward rectifying current appearing after break-in is carried by Cl^−^, as substitution of vacuolar Cl^−^ by gluconate significantly reduces its amplitude (Figure 1E, red IV). TRPY1 inward and outward cation currents were already detectable at [Ca^2+^]_cyt_ of 10 μM (Figures 2A and 2B), and Ca^2+^ non-cooperatively (Hill coefficient < 1) increased currents in a concentration-dependent manner with apparent EC_50_ values of 498 μM for the inward current and 391 μM for the outward current (Figure 2B).

### Vacuolar Ca^2+^ Inhibits TRPY1 Activity

To characterize the dependence of TRPY1 currents on vacuolar [Ca^2+^], we perfused the vacuole with 1 mM Ca^2+^ by the patch pipette. The currents activated by 1 mM [Ca^2+^]_cyt_ were significantly reduced (Figures 2C and 2D). Figure 2C shows the vacuolar [Ca^2+^]-dependent inhibition of TRPY1 currents activated by 1 mM [Ca^2+^]_cyt_ with apparent IC_50_ values of 79 μM for the inward current and 109 μM for the outward current (Figure 2D). We consistently found Hill coefficients > 1 (1.4–1.5, Figure 2D) suggesting that the channel is inhibited by the cooperative binding of two molecules of Ca^2+^. Inhibition of TRPY1 currents by vacuolar Ca^2+^ is still prominent at a pH value of 5.5 (Figure S1), considering the slightly acidic pH in the yeast vacuole (Preston et al., 1989).

Sr^2+^, Ba^2+^, and Mn^2+^ substitute for Ca^2+^ in TRPY1 activation (when present at the cytosolic site) and inhibition (when present at the vacuolar site) (Figure S2). In the absence of vacuolar Ca^2+^ ([Ca^2+^]_vac_), cytosolic concentrations of 1 mM divalent cations increased activation in the order Sr^2+^ (12%) < Mn^2+^ (15%) < Ba^2+^ (63%) with 100% current activation at 1 mM [Ca^2+^]_cyt_ (Figures S2A and S2B). At 100% current activation (1 mM [Ca^2+^]_cyt_, and 0 [Ca^2+^]_vac_), vacuolar concentrations of 1 mM divalent cations inhibited in the order Mn^2+^ (33%) < Ba^2+^ (63%) < Sr^2+^ (68%) < Ca^2+^ (96%) (Figures S2C and S2D).

### In HEK-293 Cells TRPY1 Retains Its Ca^2+^-Dependent Properties

**Figure 3:**
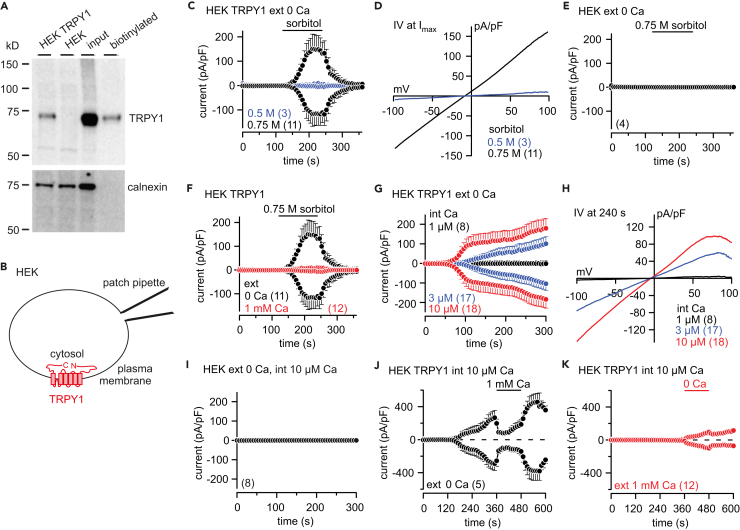
Ca^2+^-Dependent Regulation of TRPY1 Currents in Transfected HEK-293 Cells (A) Western blot of protein lysates from *YVC1* cDNA-transfected (HEK TRPY1) and non-transfected (HEK) HEK-293 cells. Biotinylation of surface proteins shows that a considerable fraction of TRPY1 present in transfected HEK-293 cells (input) resides in the plasma membrane (biotinylated). The filter was stripped and incubated in the presence of an antibody directed against the intracellular protein calnexin as control. (B) Orientation of TRPY1 (red) in the plasma membrane with N- and C-termini residing within the cytosol. (C–K) Inward and outward currents at −80 and 80 mV, extracted from 400-ms ramps (0.5 Hz) spanning from −100 to 100 mV, V_h_ = 0 mV, plotted versus time (C, E–G, and I–K) and corresponding IVs (D and H) of maximum currents (I_max_) from (C) or at 240 s from (G), activated by 0.5 or 0.75 M sorbitol (hyperosmotic shock; C–F) or indicated [Ca^2+^]_cyt_ (int Ca; buffered with BAPTA; G-K) at 0 or 1 mM external (ext) Ca^2+^ in HEK TRPY1 cells (HEK TRPY1; C, D, F–H, J, and K) or non-transfected HEK-293 cells (HEK; E and I). The bars indicate application of 0.5 or 0.75 M sorbitol in C, E, and F, or 0 and 1 mM Ca^2+^-containing bath solution in K and J, respectively. The black traces in C and F are the same. Currents and IVs are normalized to the cell size (pA/pF) and are shown as means ± SEM (C, E–G, and I–K) and means (D and H) with the number of measured cells in brackets.

In yeast, TRPY1 was shown to be also activated by hyperosmotic shock, i.e., in the presence of high extracellular concentrations of sorbitol or NaCl (Zhou et al., 2003). Hyper- or hypoosmotic shock induces shrinkage or swelling of the vacuole, respectively, which easily disrupts the whole-vacuolar patch-clamp configuration. To prove whether hyperosmotic shock-induced TRPY1 activity is also inhibited by Ca^2+^, we expressed the *YVC1* cDNA in HEK-293 cells. As shown by western blot the TRPY1 protein is present in transfected cells and a significant fraction is detectable in the plasma membrane by surface biotinylation (Figure 3A). In the plasma membrane the channel regions facing the vacuole in yeast are now facing the extracellular bath, whereas, like in yeast, the N-terminus, C-terminus, S2-S3, and S4-S5 linkers are facing the cytoplasm (Figure 3B). As shown in Figures 3C and 3D, TRPY1 currents are activated in the absence of extracellular Ca^2+^ by increasing the osmolarity in the bath by application of 0.5 and 0.75 M sorbitol, whereas no currents were detectable in non-transfected HEK-293 cells (Figure 3E). Applying 1 mM Ca^2+^ to the bath significantly reduced the TRPY1 current induced by 0.75 M sorbitol (Figure 3F, red trace) indicating that TRPY1 integrates activating and inhibitory stimuli also in HEK-293 cells.

In the absence of extracellular Ca^2+^, TRPY1 inward and outward currents in HEK-293 cells are activated by [Ca^2+^]_cyt_ (at 3 and 10 μM; Figures 3G and 3H), confirming the assumed orientation of TRPY1 in the plasma membrane. Non-transfected HEK-293 cells do not reveal any significant currents under these conditions (Figure 3I). TRPY1 currents activated by 10 μM [Ca^2+^]_cyt_ in the absence of extracellular Ca^2+^ are readily inhibited when 1 mM Ca^2+^ was added (Figure 3J). Figure 3K shows that in the presence of 1 mM external Ca^2+^, almost no TRPY1 current appeared at [Ca^2+^]_cyt_ of 10 μM, whereas after removal of external Ca^2+^ a significant current developed (Figure 3K).

### Aspartate Residues 401 and 405 Mediate the Ca^2+^-Dependent Inhibition of TRPY1

**Figure 4:**
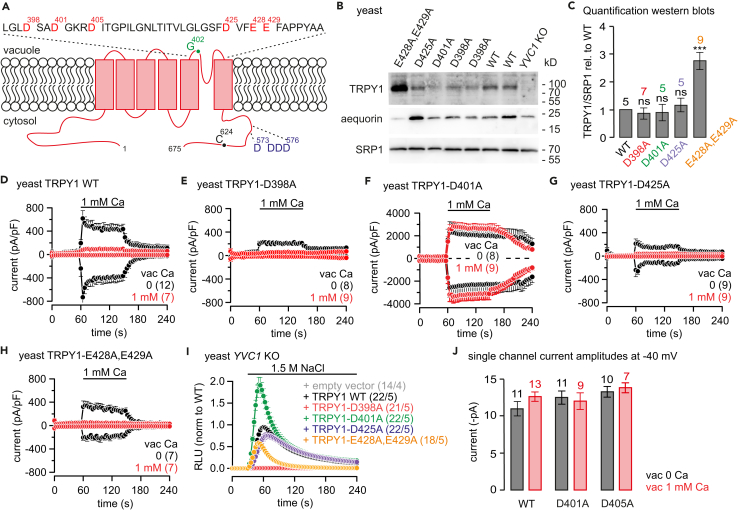
Vacuolar TRPY1 Currents in *YVC1* KO Yeast Expressing Wild-Type or Mutant *YVC1* cDNAs (A) Predicted transmembrane topology of the yeast vacuolar TRPY1 (675 amino acid residues) with negative charge cluster at the cytosolic C-terminus assumed to be responsible for TRPY1 activation (blue) (Su et al., 2009) and negatively charged residues within the S5-S6 linker facing the vacuolar lumen (red). The TRPY1_G402S_ mutant (green) is constitutively active (Zhou et al., 2007), and the TRPY1_C624S_ mutant (black) can no longer be activated by 2-mercaptoethanol (Hamamoto et al., 2018). (B) Western blot of protein lysates from non-transformed yeast (*YVC1* KO) or *YVC1* KO yeast transformed with wild-type (WT) and mutant *YVC1* cDNAs (as indicated). Filter was stripped and incubated with antibodies for aequorin (middle) and SRP1 (serine-rich protein; bottom) as controls. (C) Antibody stain intensities of TRPY1 proteins in (B) were quantified relative to the antibody stain of the endogenous serine-rich protein 1 (SRP1) and normalized to the WT TRPY1/SRP1 ratio (summary of five independent western blots). One-way analysis of variance (ANOVA): not significant (ns), ^∗∗∗^ p<0.001 compared to WT. (D–H) Whole-vacuole currents at −80 and 80 mV extracted from 200-ms ramps (0.5 Hz) spanning from 150 to −150 mV, V_h_ = 0 mV, plotted versus time, activated by cytosolic Ca^2+^ (1 mM) at 0 (black) or 1 mM (red) vacuolar (vac, patch pipette) Ca^2+^ in *YVC1* KO cells expressing WT ( D) or mutant (E–H) *YVC1* cDNAs. (I) Changes of cytosolic Ca^2+^ challenged by 1.5 M NaCl (hyperosmotic shock) monitored in transformed *YVC1* KO yeast cells (as in D–H) as relative luminescence units (RLU). Bars indicate application of 1 mM Ca^2+^ (D–H) or 1.5 M NaCl (I). (J) Single-channel current amplitudes of TRPY1_WT_, TRPY_D401A_, and TRPY1_D405A_ in the absence and presence of vacuolar 1 mM Ca^2+^, analyzed at −40 mV from current traces of voltage ramps and voltage steps in whole vacuoles and excised (outside-out) vacuolar patches. Single channels of TRPY1_D405A_ were analyzed from yeast cells transfected with single-copy *pRS316* CEN plasmids without its own promoter (see Figure S4C). Currents are normalized to the size of the vacuole (pA/pF) and shown as means ± SEM with number of measured vacuoles (D–H) or the number of experiments (x) from y independent transformation (x/y; (I) indicated in brackets. Single-channel current amplitudes in (J) are shown as means ± SEM with number of independent experiments indicated. One-way analysis of variance (ANOVA) revelead no differences. See also Figures S3 and S4.

The mechanism of the inhibition of TRPY1 currents by vacuolar Ca^2+^ in yeast or extracellular Ca^2+^ in HEK-293 cells was not known. We noticed six negatively charged acidic residues in the S5-S6 linker contributing to the TRPY1 pore, D398, D401, D405, D425, E428, and E429 (Figure 4A), which we replaced by alanine residues. The cDNAs of all mutants and of wild-type TRPY1 (plasmids see Table 1) were expressed in *YVC1* KO yeast (Chang et al., 2010). Wild-type and mutant proteins are detectable in western blot (Figure 4B). Only the transformation of yeast with plasmid *pRS316-TRPY1*_*D405A*_ did not yield any colonies. Figures 4D–4H show currents of wild-type TRPY1 (Figure 4D) and of the TRPY1 mutants, expressed in *YVC1* KO yeast (Figures 4E–4H), activated by 1 mM [Ca^2+^]_cyt_ in the absence (Ca 0) or presence of 1 mM vacuolar Ca^2+^. As shown for vacuoles of wild-type yeast cells (Figure 2) 1 mM [Ca^2+^]_cyt_ activates TRPY1 in the absence (Figure 4D, black trace), but not in the presence of vacuolar Ca^2+^ (Figure 4D, red trace), whereas TRPY1_D398A_ failed to yield currents even in the absence of vacuolar Ca^2+^ (Figure 4E). The Ca^2+^-mediated outward current detectable in the absence of vacuolar Ca^2+^ most probably reflects changes of the vacuolar chloride conductance (see Figure 1). Expression of TRPY1_D401A_ yielded a small constitutive current (Figure S3A, green trace), which could be significantly enhanced by 1 mM [Ca^2+^]_cyt_, leading to current amplitudes more than five times higher than mediated by wild-type TRPY1 (Figure 4F, black trace), but currents were no longer inhibited by vacuolar Ca^2+^ (Figure 4F, red trace). Beside smaller current amplitudes, TRPY1_D425A_ and TRPY1_E428A,E429A_ revealed the same Ca^2+^ dependence as TRPY1 wild-type (Figures 4G and 4H). Ca^2+^ imaging experiments obtained after expressing the cytosolic luminescent Ca^2+^ reporter aequorin in intact yeast confirmed the patch-clamp data (Figure 4I). TRPY1_D401A_ induced the highest cytosolic Ca^2+^ increase upon hyperosmotic shock, whereas TRPY1_D398A_ did not respond at all. Current-voltage relationships and statistics of whole-vacuole current amplitudes and yeast cytosolic Ca^2+^ signals are summarized in Figure S3. The reversal potential of vacuolar currents did not vary between TRPY1 mutants and wild-type, arguing against changes of ion permeability.

**Table 1:** TRPY1 Plasmids Generated in the Laboratories of the Authors and Used in This Study

Plasmid	TRPY1 Construct
Backbone: Yeast single-copy centromeric pRS316 vector	
pGS2062	TRPY1 wild-type
pSB276	TRPY1_D398A_
pSB277	TRPY1_D401A_
pSB278	TRPY1_D405A_
pSB279	TRPY1_D425A_
pSB280	TRPY1_E428A,E429A_
Backbone: Bicistronic eukaryotic expression (HEK-293 cells)	
pCAGGS-YVC1-IRES-GFP	TRPY1 wild-type
pCAGGS-YVC1_D398A_-IRES-GFP	TRPY1_D398A_
pCAGGS-YVC1_D401A_-IRES-GFP	TRPY1_D401A_
pCAGGS-YVC1_D405A_-IRES-GFP	TRPY1_D405A_
pCAGGS-YVC1_D425A_-IRES-GFP	TRPY1_D425A_
pCAGGS-YVC1_E428A,E429A_-IRES-GFP	TRPY1_E428A,E429A_

The *YVC1* gene encoding wild-type TRPY1 (NCBI accession number NM_001183506.1) was subcloned into the single-copy centromeric pRS316 vector (Sikorski and Hieter, 1989) under control of its own promoter and termination sequence. For some transformations (Figures S4A–S4C) the latter sequences were omitted. The eukaryotic expression vector pCAGGS has been described (Wissenbach et al., 2001).

Wild-type and mutant *YVC1*-transformed *YVC1* KO yeast showed different amplitudes of vacuolar currents and cytosolic Ca^2+^ signals: D401A revealed larger currents and higher [Ca^2+^]_cyt_ than wild-type TRPY1, whereas for TRPY1_D425A_ and TRPY1_E428A,E429A_, currents and changes of [Ca^2+^]_cyt_ were smaller (Figures 4D–4I). Western blots revealed similar amounts of TRPY1 protein in yeast transformed with wild-type, D398A, D401A, and D425A *YVC1*, relative to the endogenous SRP1, used as a control (Figure 4C). The higher amount of TRPY1_E428A,E429A_ protein (Figure 4C) not mirrored by larger currents (Figure 4H) and higher [Ca^2+^]_cyt_ (Figure 4I) might indicate that only part of the protein detectable in western blot is targeted to the vacuolar membrane.

Transformation of yeast with *pRS316-YVC1*_*D405A*_ did not yield any colonies. To yield lower expression levels to prevent delirious effects of overexpression, we cloned the *YVC1* gene (wild-type, *YVC1*_*D401A*_, and *YVC1*_*D405A*_) in single-copy *pRS316* CEN plasmids without its own promoter. Under these conditions, cytosolic Ca^2+^-induced vacuolar currents were recorded from *YVC1* wild-type and *YVC1*_*D401A*_- and *YVC1*_*D405A*_-transformed *YVC1* KO yeast (Figures S4A–S4C). In contrast to wild-type TRPY1 (Figure S4A), the currents induced by cytosolic Ca^2+^ remained in the presence of 1 mM vacuolar Ca^2+^ in TRPY1_D401A_ (Figure S4B; note the spontaneous current after break-in) and TRPY1_D405A_ (Figure S4C).

Beside a smaller current amplitude of TRPY1_D405A_ at 40 mV in the absence of vacuolar 1 mM Ca^2+^ (see Figure S4E), neither the mutations D401A and D405A nor the presence of vacuolar 1 mM Ca^2+^ significantly affected single-channel current amplitudes of TRPY1 analyzed at −40 and 40 mV (Figures 4J and S4E, respectively; example traces are shown in Figure S4D). Single-channel current amplitudes of TRPY1_D405A_ were analyzed from yeast cells transfected with single-copy *pRS316* CEN plasmids without its own promoter (see above). The unchanged single-channel current amplitude (Figures 4J and S4E) but significantly reduced whole-vacuolar current amplitude (Figure 4D) in wild-type TRPY1 suggests a prominent decrease of the open probability in the presence of vacuolar 1 mM Ca^2+^.

**Figure 5:**
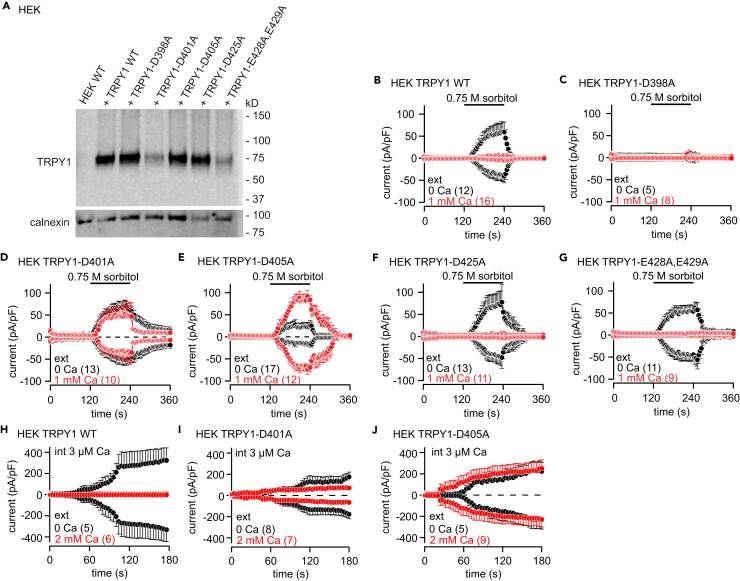
Wild-Type and Mutant TRPY1 Currents Recorded from HEK-293 Cells (A) Western blot of protein lysates from non-transfected HEK-293 cells (HEK WT) and HEK-293 cells transfected with the *YVC1* wild-type (+ TRPY1 WT) and the mutant *YVC1* cDNAs as indicated. The filter was stripped and incubated with an antibody for calnexin as loading control (bottom panel). (B–J) Inward and outward currents at −80 and 80 mV extracted from 400-ms ramps (0.5 Hz) spanning from −100 to 100 mV, V_h_ = 0 mV, plotted versus time, activated by 0.75 M sorbitol (bar, hyperosmotic shock; B–G) or 3 μM cytosolic Ca^2+^ (H–J) at 0 (black), 1 (B–G), or 2 mM (H–J) external Ca^2+^ (red) in HEK-293 cells transiently transfected with WT (B and H) or mutant (C–G, I, and J) *YVC1* cDNAs. Currents are normalized to the cell size (pA/pF). Data are shown as means ± SEM with the number of measured cells in brackets. See also Figure S5.

After expression of wild-type and mutant *YVC1* cDNAs (plasmids see Table 1) in HEK-293 cells, all proteins including TRPY1_D405A_ were detectable (Figure 5A), and we recorded hyperosmotic shock (Figures 5B–5G) as well as cytosolic Ca^2+^-induced currents (Figures 5H–5J). Current-voltage relationships and statistics of whole-cell current amplitudes are summarized in Figure S5. Compared with wild-type TRPY1 (Figure 5B) hyperosmotic shock did not initiate TRPY1_D398A_ currents (Figure 5C), but the TRPY1_D425A_ (Figure 5F) and TRPY1_E428A,E429A_ mutants (Figure 5G) behaved like wild-type TRPY1, very similar as observed in the yeast vacuole (Figure 4). Like in yeast vacuoles TRPY1_D401A_ yielded spontaneous currents (Figures 5D and S5A, green trace), and, compared with wild-type TRPY1 (Figures 5B and 5H), extracellular Ca^2+^ (1 and 2 mM, respectively) did not inhibit hyperosmotic shock and cytosolic Ca^2+^-induced activity of the mutant channels TRPY1_D401A_ and TRPY1_D405A_ (Figures 5D, 5I, 5E, and 5J). The latter mutant induced even larger current amplitudes upon hyperosmotic shock in the presence of extracellular Ca^2+^. Current-voltage relationships and statistics of current amplitudes are summarized in Figure S5.

**Figure 6:**
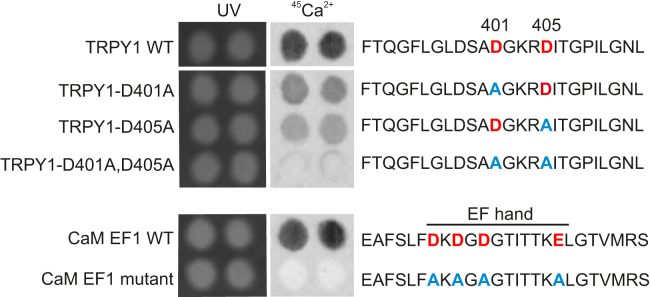
Ca^2+^ Binding of TRPY1 Autoradiographs of ^45^Ca^2+^ binding by the 25-mer peptides derived from wild-type (amino acid residues F_390_ to L_414_) and D401A and D405A mutant S5-S6 linkers of TRPY1 (upper panel) and by the 25-mer peptides representing wild-type and mutant calmodulin EF hand 1 (CaM EF1, lower panel) as control (two out of four performed blots are shown). Peptides were synthesized and spotted onto hardened cellulose membranes (about 16 nmoles per spot) and incubated with 1.5 μM (1 mCi/L) ^45^Ca^2+^. Amounts of peptides spotted were additionally estimated by UV absorption at 312 nm. Aspartate (D) residues are marked in red and alanine (A) residues replacing aspartates are marked in blue. See also Figure S6.

To prove whether residues D401 and D405 are required for Ca^2+^ binding 25-amino-acid (25-mer) peptides derived from wild-type (amino acid residues F_390_ to L_414_) and D401A and D405A mutant S5-S6 linkers of TRPY1 were synthesized and spotted onto hardened cellulose membranes and incubated with ^45^Ca^2+^ (Figure 6). The wild-type peptide significantly binds ^45^Ca^2+^, whereas the TRPY1_D401A_ and TRPY1_D405A_ peptides showed less and the double mutant (TRPY1_D401A,D405A_) no detectable ^45^Ca^2+^ binding. As control, we spotted a 25-mer peptide representing the first EF hand (EF1) of mammalian calmodulin (CaM; Figure 6). The ^45^Ca^2+^ binding by EF1 was completely abolished when its glutamate residues were replaced by alanine residues (Figure 6). The amounts of ^45^Ca^2+^ bound by EF1 and TRPY1_F390-L414_ are very similar, as estimated by the signals' intensities. Considering equal amounts of spotted 25-mer peptides (about 16 nmoles per spot), additionally estimated by UV absorption at 312 nm (Figure 6), close apparent binding affinities of the peptides for Ca^2+^ might be assumed. At physiological salt concentration, the non-cooperative Ca^2+^ binding affinity of the isolated CaM EF1 was estimated to be about 125 μM (Ye et al., 2005), which is very close to the value of 79–109 μM measured here for vacuolar Ca^2+^-dependent inhibition of TRPY1 (Figure 2D).

## Discussion

We performed whole-vacuole patch-clamp recordings and Ca^2+^ imaging experiments in yeast to study the Ca^2+^ dependence of TRPY1 using as controls a *YVC1*-deficient yeast strain (Chang et al., 2010) and HEK-293 cells heterologously expressing the *YVC1* cDNA as functional TRPY1 channels in the plasma membrane. We show that inhibition of vacuolar TRPY1 is cooperatively mediated by the vacuolar [Ca^2+^] (IC_50_ 79–109 μM), that it requires two aspartate residues (D401 and D405) in the S5-S6 linker facing the vacuolar lumen, and that these aspartate residues are directly involved in Ca^2+^ binding. The yeast vacuolar TRP cation channel TRPY1 may be assumed as a progenitor of TRP channels present in mammals, flies, and worms and underlies cation movements across the yeast vacuolar membrane. Additional TRP-related proteins in *Saccharomyces cerevisiae* like FLC2, encoded by the *YAL053W* gene, seem to be involved in endoplasmic reticulum Ca^2+^ release (Rigamonti et al., 2015). TRPY1 is activated by osmotic stress (Batiza et al., 1996, Denis and Cyert, 2002, Zhou et al., 2003), reducing agents (Bertl and Slayman, 1990, Wada et al., 1987), aromatic compounds like indole (John Haynes et al., 2008), and cytosolic Ca^2+^ (Bertl and Slayman, 1990, Wada et al., 1987). In the presence of DTT the EC_50_ values of TRPY1 activation by cytosolic Ca^2+^ are 498 μM (inward current) and 391 μM (outward current) with Hill coefficients < 1. Reducing agents such as DTT or β-mercaptoethanol lower the [Ca^2+^]_cyt_ required for channel activation (Bertl and Slayman, 1990, Bertl and Slayman, 1992), and the cysteine residue 624 within TRPY1's C terminus facing the cytosol (Figure 4A) has been suggested to be involved in this process (Hamamoto et al., 2018). Reducing agents may mimic the redox environment in the yeast cytosol (Carpaneto et al., 1999, Lopez-Mirabal and Winther, 2008, Palmer et al., 2001), which might contribute to TRPY1 activation under certain conditions. Vacuolar TRPY1 currents are already activated at [Ca^2+^]_cyt_ of 10 μM of cytosolic Ca^2+^ (Figure 2 and Bertl et al., 1992b, Bertl and Slayman, 1990, Bertl and Slayman, 1992), but the yeast [Ca^2+^]_cyt_ has been estimated to be in the range of 260 nM (Halachmi and Eilam, 1989). Probably, hyperosmotic shock represents the initial physiological stimulus in intact yeast, which increases cytosolic Ca^2+^, which in turn facilitates TRPY1 activity. Palmer et al. (Palmer et al., 2001) suggested that the cytosolic activation of TRPY1 is Ca^2+^ specific, but we also detected activation by the alkaline earths Ba^2+^ > Sr^2+^ and by the transition metal ion Mn^2+^ (Figure S2).

High [Ca^2+^] within the vacuolar lumen presumably inhibits TRPY1 currents (Zhou et al., 2003), and 1 mM Ca^2+^ added to the bath eliminates TRPY1 single-channel currents in inside-out excised vacuolar patch-clamp recordings (Hamamoto et al., 2018). The present study shows that vacuolar Ca^2+^, as well as Sr^2+^ and Ba^2+^, strongly inhibit TRPY1 activity and that the amino acid residues D401 and D405 located in the vacuolar loop between S5 and S6 (Figure 4A) are crucial for Ca^2+^-dependent inhibition and direct Ca^2+^ binding. Mutation of the nearby G402 (Figure 4A) renders TRPY1 constitutively active (Zhou et al., 2007), indicating the significance of this area for proper gating. Hamamoto et al. described that luminal Zn^2+^ increased TRPY1 currents in vacuolar excised patches (Hamamoto et al., 2018). Zn^2+^ might be attracted to a single negatively charged residue and thereby prevent efficient coordination of Ca^2+^. In mouse TRPV3 and TRPV4, corresponding residues D641 and D682, respectively, were shown to be key residues for extracellular Ca^2+^-mediated channel inhibition (Voets et al., 2002, Watanabe et al., 2003, Xiao et al., 2008), and human TRPV1 D646 was suggested to be a high-affinity binding site for cations (Garcia-Martinez et al., 2000). However, D646 and D682 are part of the selectivity filter in TRPV1 and TRPV4, respectively (Deng et al., 2018, Liao et al., 2013), and, by sequence comparison, rather align to TRPY1_D425_ (Figure S6), whereas TRPY1_D401_ and TRPY1_D405_ are located in the vacuolar vestibule of TRPY1's ion-conducting pore. The residues E600, D601, E610, and E648 are involved in pH-dependent modulation of human TRPV1 (Jordt et al., 2000), with TRPV1_D601_ aligning to TRPY1_D401_ (Figure S6A). Although nothing is known about external Ca^2+^-mediated inhibition of TRPV1 and TRPV6, their S5-S6 linkers appear closer related to TRPY1 than TRPV3, TRPV4, TRPV2, and TRPA1 by sequence alignment (Figure S6A). TRPV6 residues D517 and E518, located in the upper vestibule and aligning with TRPY1_D401_, are shown to bind and guide Ca^2+^ into the channel pore of TRPV6 (Singh et al., 2017).

The structure of TRPY1 is not yet available. Therefore, we aligned the structure of TRPY1 according to the published structure of *Xenopus tropicalis* TRPV4 (Deng et al., 2018), which is inhibited by Ca^2+^ from the extracellular side (see above; Figures S6B and S6C). Our data show that TRPY1 D401 and D405 are decisive for the Ca^2+^-dependent inhibition of the channel. Both residues, D401 and D405, also highly conserved in TRPY homologs of other fungi (Ihara et al., 2014), are part of the outer pore domain of the channel and orientated to the lumen of the vacuole and are directly involved in Ca^2+^ binding. The alignment suggests that binding of Ca^2+^ to residues D401 and D405 might result in a reorientation of residues I455 and Y458, possibly involved in forming the lower gate of TRPY1, and thereby narrowing or occluding the lower gate (Figure S6B). According to the model, replacing D401 and D405 by alanine residues disables Ca^2+^ binding and thus prevents such structural changes in the presence of vacuolar Ca^2+^ (Figure S6C). As single-channel current amplitudes of TRPY1 are not affected (Figure 4J), we assume that Ca^2+^ binding to D401 and D405 affects the open probability by rearranging the main S6 segment, stabilizing a closed inactivated conformation of the channel by narrowing and occluding the lower gate.

The physiological pH value in yeast vacuoles is slightly acidic (Pearce et al., 1999, Preston et al., 1989). Vacuolar pH 6.0 does not alter the mechanosensitivity of TRPY1 (Zhou et al., 2003), whereas the current activated by cytosolic Ca^2+^ was reduced at a vacuolar pH 5.5, but the vacuolar Ca^2+^-dependent inhibition remained (Figure S1). The mammalian TRP channels TRPML1, 2, and 3 are located in endolysosomes, and thus exert their functions also in a cytoplasmic organelle environment as TRPY1. TRPML channels are involved in the release of Ca^2+^ and Fe^2+^ into the cytosol (Cheng et al., 2010), and TRPML1 has been shown to be regulated by lysosomal Ca^2+^ and pH (Cantiello et al., 2005, Li et al., 2017). Like for wild-type TRPY1 a significant fraction of the constitutively active TRPML1 mutant, TRPML1_V432P_, can be expressed in the plasma membrane of HEK-293 cells. Extracellular Ca^2+^ inhibits TRPML1_V432P_ channel activity with an apparent IC_50_ of 270 μM (Li et al., 2017), which is in the range of the vacuolar [Ca^2+^], which inhibits TRPY1 (Figure 2). External (lysosomal) acidification reduced the Ca^2+^-dependent inhibition of TRPML1_V432P_, and an electronegative luminal pore domain was indicated as a unique hallmark of TRPML1's interaction site for lysosomal Ca^2+^ and H^+^ (Li et al., 2017).

The yeast vacuolar Ca^2+^ concentration was estimated to be 1.3 mM (Halachmi and Eilam, 1989, Iida et al., 1990) and the vacuole was assumed to function as a Ca^2+^ store with TRPY1 as Ca^2+^ release channel (Denis and Cyert, 2002). According to our results TRPY1 currents would be inhibited at 1.3 mM Ca^2+^, but a considerable amount of Ca^2+^ is bound by vacuolar polyphosphate (Dunn et al., 1994) and therefore most probably not available for channel inhibition. The remaining vacuolar free [Ca^2+^] of ∼30 μM (Dunn et al., 1994) would allow significant TRPY1 currents (Figure 2). Considering that monovalent cations K^+^ and Na^+^ are the major charge carriers used to record TRPY1 currents in the yeast vacuole and in HEK-293 cells, Ca^2+^ storage and release may not represent major functions of vacuoles and TRPY1 channels. Instead, upon hypertonic shock, which activates TRPY1, cation efflux from the vacuole might provide osmolytes to the cytosol and thereby counteracts water deprivation. The vacuolar Ca^2+^-dependent inhibition of TRPY1 might protect the yeast cell from cytosolic Ca^2+^ overload after hyperosmotic-shock-induced channel activation and increase of the vacuolar [Ca^2+^] subsequent to loss of vacuolar water.

### Limitations of the Study

We studied the Ca^2+^-dependent modulation of the yeast vacuolar TRP channel and identified two aspartate residues in the outer vestibule as essential for Ca^2+^ binding and Ca^2+^-dependent inhibition. The structure of TRPY1 is not yet known, and we aligned the S5, pore linker, and S6 structure of TRPY1 according to the published structure of *Xenopus* TRPV4. Thus the mechanism we discuss for the Ca^2+^-dependent inhibition of TRPY1 reflects our experimental data along with a TRPY1 model, obtained due to an alignment to the available structure of another TRP protein. The suggested scenario awaits further support by structural data of TRPY1.

## Methods

All methods can be found in the accompanying Transparent Methods supplemental file.

## Supplemental Information

Document S1. Transparent Methods and Figures S1–S6
