# Supplementary material for: Identification of Inhibitory Ca2+ Binding Sites in the Upper Vestibule of the Yeast Vacuolar TRP Channel
Source: iScience. 2018 Dec 4;11:1–12. doi: 10.1016/j.isci.2018.11.037 (PMC6299153; doi:10.1016/j.isci.2018.11.037)
Supplement: Document S1. Transparent Methods and Figures S1–S6 [file mmc1.pdf]

ISCI, Volume *11*

Supplemental Information

Identification of Inhibitory Ca^{2+} Binding Sites in the Upper Vestibule of the Yeast Vacuolar TRP Channel

Mahnaz Amini, Hongmei Wang, Anouar Belkacemi, Martin Jung, Adam Bertl, Gabriel Schlenstedt, Veit Flockerzi, and Andreas Beck

Figure S1 Vacuolar Ca^{2+}-dependent inhibition of TRPY1 at vacuolar pH 5.5, related to Figure 2

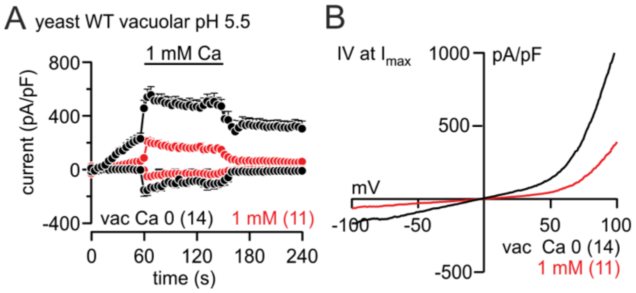

(**A**) Whole-vacuole currents at -80 and 80 mV extracted from 200 ms ramps (0.5 Hz) spanning from 150 to -150 mV, V_h=0 mV, plotted versus time, activated by cytosolic Ca^{2+} (1 mM, indicated by the bar) in the absence (black) and presence (red) of 1 mM Ca^{2+} (vacuolar, applied by the patch pipette) at a vacuolar pH of 5.5. (**B**) Current-voltage relationships (IVs) of maximum Ca^{2+}-induced (I_{max}) whole-vacuole currents in **A**. Currents are shown as means ± S.E.M. and IVs as means with the number of measured vacuoles in brackets.

Figure S2 Effects of divalent cations in the cytosol and in the vacuole on TRPY1 currents, related to Figure 2

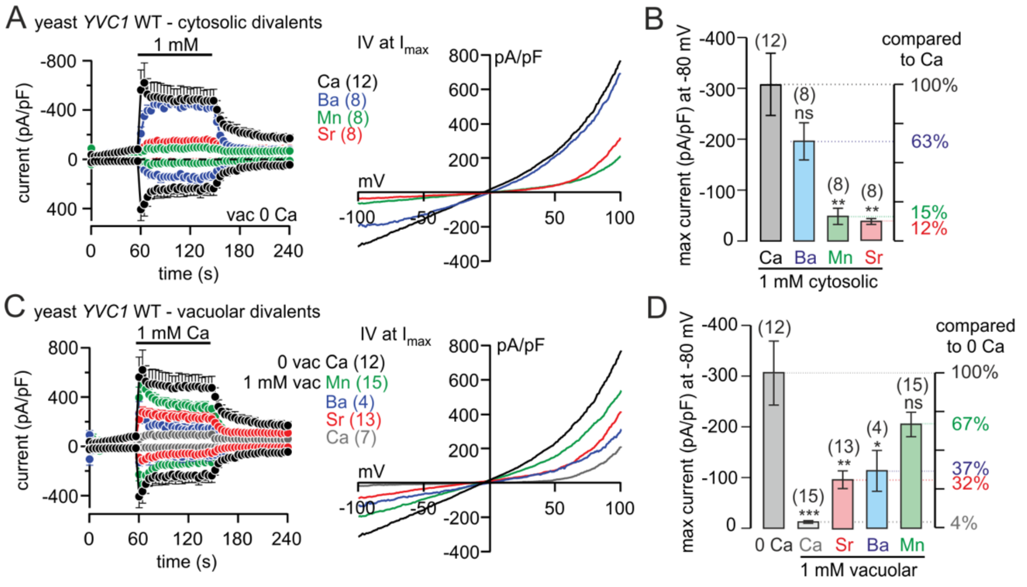

Whole-vacuole currents at -80 and 80 mV extracted from 200 ms ramps (0.5 Hz) spanning from 150 to -150 mV, V_h=0 mV, plotted versus time, activated by 1 mM cytosolic divalent cations (Ca^{2+}, Ba^{2+}, Mn^{2+}, Sr^{2+}, application indicated by the bar) in the absence of vacuolar Ca^{2+} (**A**, left) or activated by 1 mM cytosolic Ca^{2+} (application indicated by the bar) in the absence (black) and presence of 1 mM vacuolar Ca^{2+}, Ba^{2+}, Mn^{2+} or Sr^{2+} (**C**, left) in yeast *YVC1* KO cells expressing the wild-type *YVC1* cDNA. (Black traces in **A** and **C** are the same). The corresponding current-voltage relationships (IVs) are shown next to the currents in **A** and **C**. (**B**, **D**) Statistics of the current amplitudes at -80 mV corresponding to the currents shown in **A** and **C**. All currents are normalized to the size of the vacuole (pA/pF) and are shown as means ± S.E.M. (A, B, C, D), IVs depict means (A, C). The number in brackets denote the number of measured vacuoles. One-way analysis of variance (ANOVA): not significant (ns), * $p<0.05$, ** $p<0.01$, *** $p<0.001$.

Figure S3 TRPY1 wild-type and mutants in yeast vacuoles, related to Figure 4

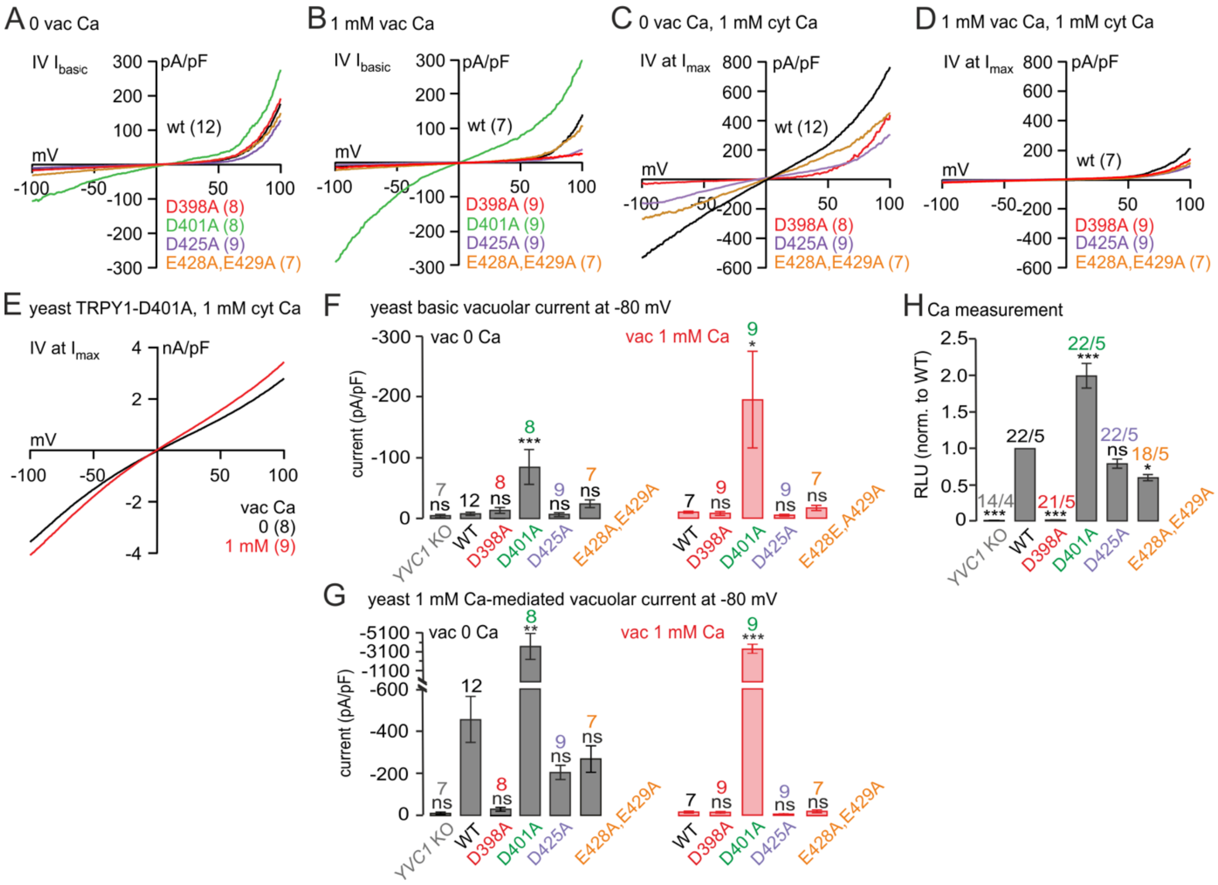

Current-voltage relationships (IVs; **A**-**E**) and current amplitudes (**F**, **G**) correspond to the data shown in Figures 4D-H): Current-voltage relationships (IVs) of the basic currents after break-in (I_{basic}; **A**, **B**) and maximum activated whole-vacuole currents (I_{max}; **C**-**E**) at 1 mM $[Ca^{2+}]_{cyt}$ in the absence (**A**, **C**, **E**) or presence (**B**, **D**, **E**) of vacuolar Ca^{2+} in yeast *YVC1* KO cells expressing *YVC1* wild-type or mutant cDNAs (Figure 4D-H). IVs are extracted from 200 ms voltage ramps spanning 150 to -150 mV, V_h=0 mV. **F** and **G** depict the statistics of the basic (**F**) and maximal Ca^{2+}-activated (**G**) currents at -80 mV from the experiments in Figure 4D-H in the absence (left, black) and presence (right, red) of 1 mM vacuolar Ca^{2+}. IVs are normalized to the cell size (pA/pF) and are shown as means; bars depict means ± S.E.M. with number of measured cells in brackets. (**H**) Statistics of the Ca^{2+} signals measured by relative luminescence units (RLU) in Figure 4I shown as means ± S.E.M. with the number of experiments (x) from y transformations (x/y). One-way analysis of variance (ANOVA): not significant (ns), * $p<0.05$, ** $p<0.01$, *** $p<0.001$ compared to WT.

Figure S4 Vacuolar currents in *YVC1* KO yeast expressing wild-type or mutant *YVC1* cDNAs (low expression levels) and single channel currents, related to Figure 4

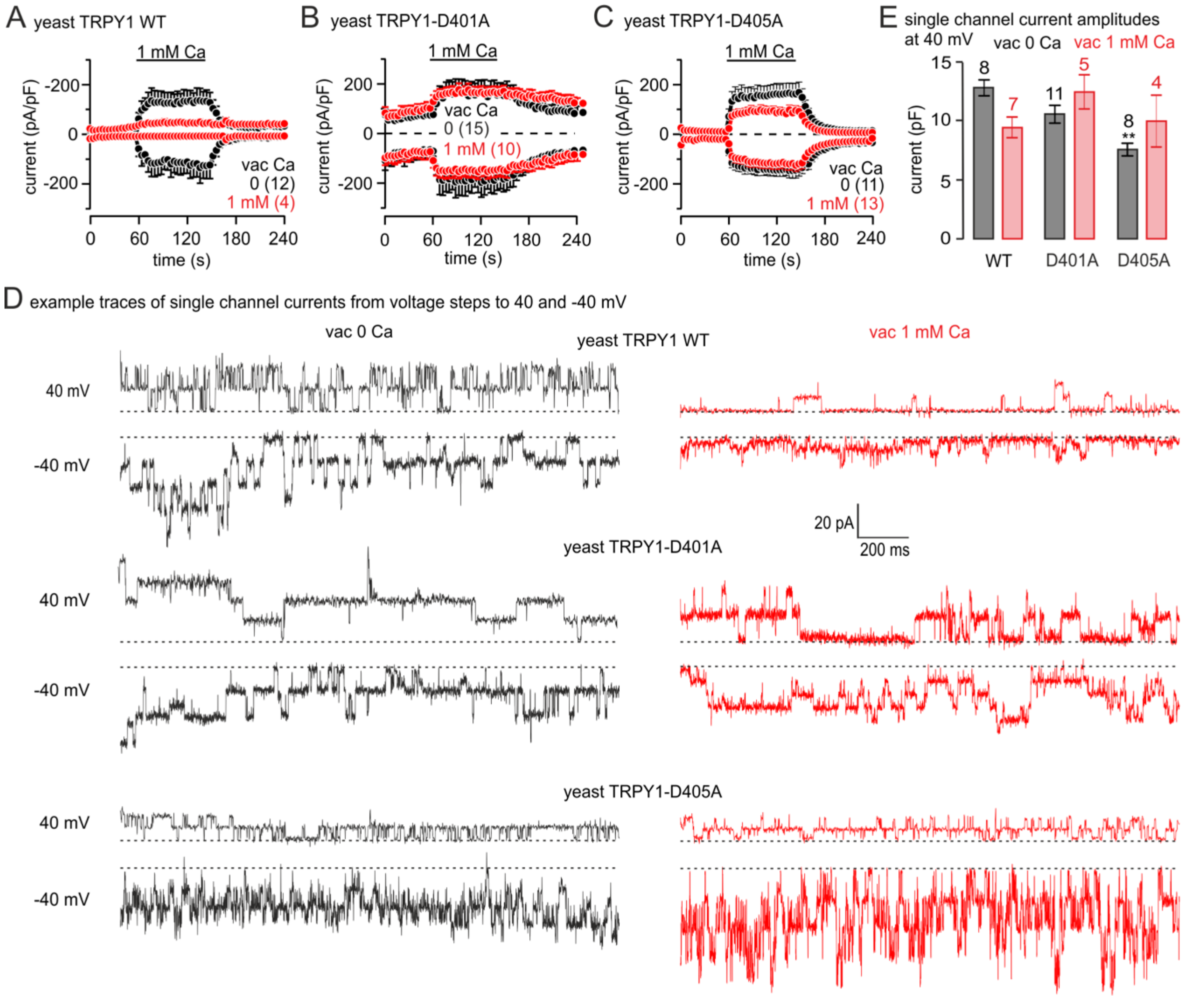

(**A**-**C**) Whole-vacuole currents at -80 and 80 mV extracted from 200 ms ramps (0.5 Hz) spanning from 150 to -150 mV, V_h=0 mV, plotted vs. time, activated by cytosolic 1 mM Ca^{2+} at 0 (black) or 1 mM (red) vacuolar (patch pipette) Ca^{2+} in yeast *YVC1* KO cells expressing TRPY1 wild type (WT; **A**), D401A (**B**) or D405A (**C**) cDNAs. (Plasmids lacking YVC1 promotor and termination sequence => low expression level of YVC1 WT and mutants => low current amplitudes compared to Figure 4). Bars indicate application of 1 mM Ca^{2+}. Currents are normalized to the cell size (pA/pF) and are shown as means ± S.E.M.. The number in brackets denote the number of measured cells (x). (**D**) Representative current traces from voltage steps to 40 mV and -40 mV applied to excised (outside-out) vacuolar patches of yeast expressing $TRPY1_{WT}$, $TRPY1_{D401A}$ and $TRPY1_{D405A}$ in the absence (left) and presence (right) of 1 mM vacuolar Ca^{2+} (dashed lines = no channels open). (**E**) Single channel current amplitudes of $TRPY1_{WT}$, $TRPY_{D401A}$ and $TRPY1_{D405A}$ in the absence and presence of vacuolar 1 mM Ca^{2+}, analyzed at 40 mV from current traces of voltage steps in whole-vacuoles and excised (outside-out) vacuolar patches. Single channels of $TRPY1_{D405A}$ were analyzed from yeast cells transfected with single copy *pRS316* CEN plasmids without its own promoter (see C). Current amplitudes in E are shown as means ± S.E.M. with number of independent experiments indicated. One-way analysis of variance (ANOVA): p<0.05, ** compared to WT in the absence of vacuolar Ca^{2+}.

Figure S5 TRPY1 wild-type and mutants in HEK-293 cells, related to Figure 5

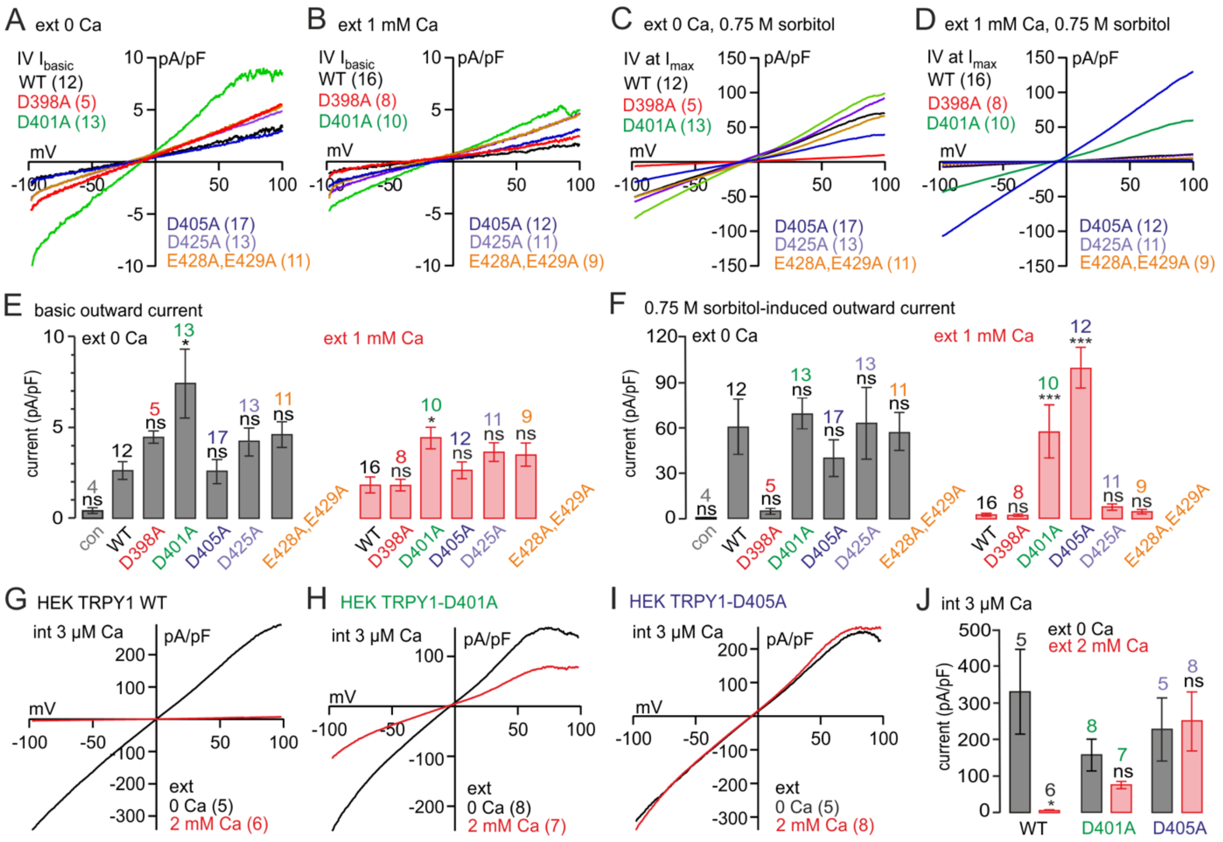

Current-voltage relationships (IVs; **A**-**D** and **G**-**I**) and current amplitudes (**E**-**F** and **J**) correspond to the data shown in Figures 5B-G and H-J, respectively: Current-voltage relationships (IVs) of the basic current after break-in (I_{basic}; **A**, **B**) and maximum 0.75 M sorbitol-induced (I_{max}; **C**, **D**) whole-cell currents in the absence (**A**, **C**) or presence (**B**, **D**) of extracellular 1 mM Ca^{2+} in HEK cells mock-transfected (empty vector; con, control), and transfected with wild-type or mutant *YVC1* cDNAs (as in Figures 5B-G). (**G**-**I**) Current-voltage relationships of 3 µM cytosolic Ca^{2+}-induced whole-cell currents in the absence (black) or presence (red) of extracellular Ca^{2+} in HEK cells transfected with wild-type or mutant *YVC1* cDNAs (as in Figures 5H-J). IVs are extracted from 400 ms voltage ramps spanning from -100 to 100 mV, V_h=0 mV, normalized to the cell size (pA/pF) and shown as means. **E** and **F** depict the statistics of the basic (**E**) and maximal 0.75 M sorbitol-induced (**F**) currents at 80 mV from the experiments in Figures 5B-G and **J** the statistics of the 3 µM cytosolic Ca^{2+}-induced current (180 s after break-in) in Figures 5H-J in the absence (left, black) and presence (right, red) of 1 mM (**E**, **F**) or 2 mM (**J**) extracellular Ca^{2+}. Bars show means ± S.E.M.. The number in brackets denote the number of measured cells. One-way analysis of variance (ANOVA): not significant (ns), * p<0.05, *** p<0.001 compared to WT (E, F) or compared to 0 ext. Ca^{2+} (J).

Figure S6 S5-S6 linker sequence alignment and structural modelling of TRPY1, related to Figure 6

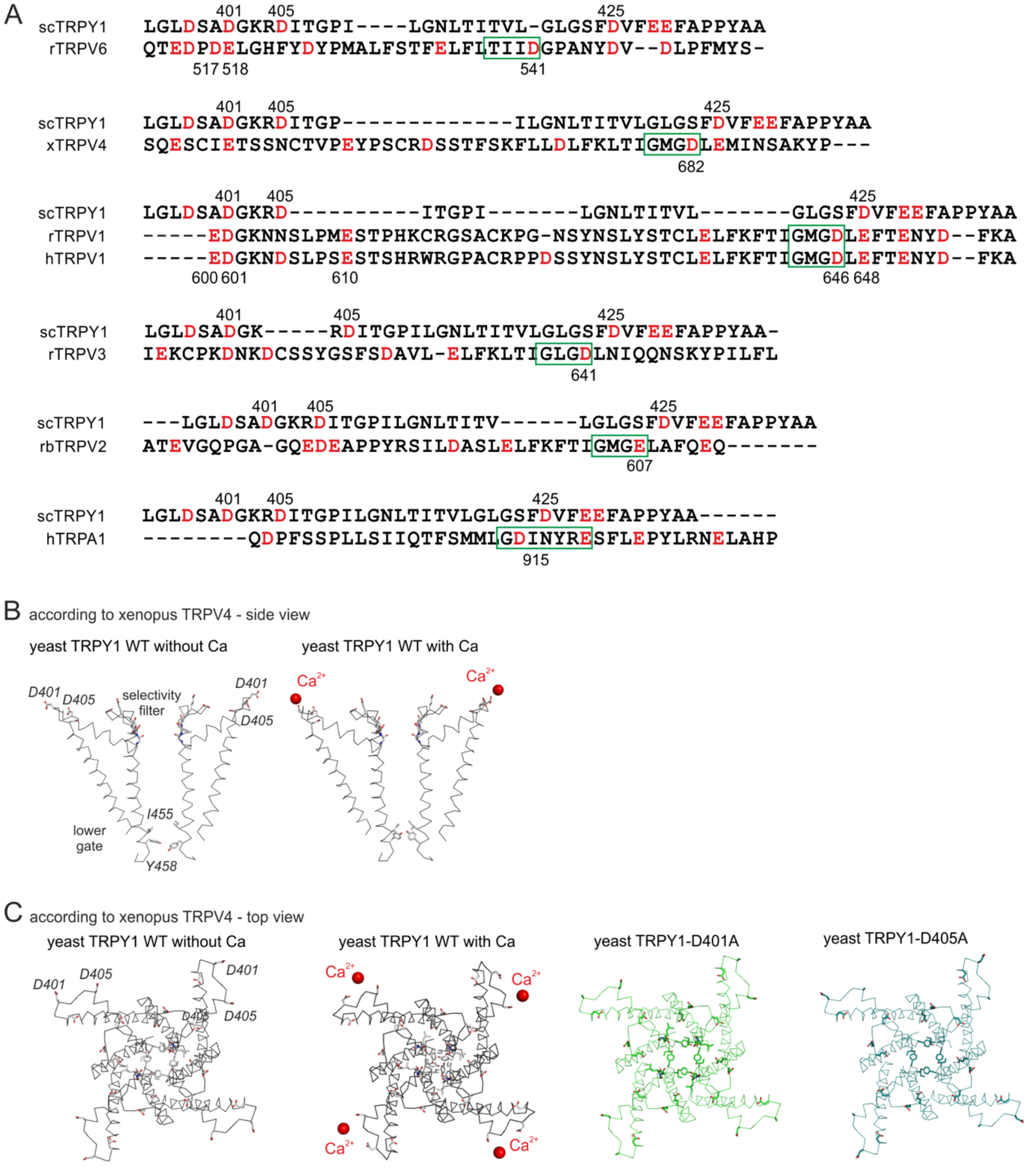

(**A**) The S4-S5 linker sequence of *Saccharomyces cerevisiae* TRPY1 (scTRPY1) and the S4-S5 linker sequences defined by the structures of rat TRPV6 (rTRPV6) (Singh et al., 2017), xenopus TRPV4 (xTRPV4) (Deng et al., 2018), rat and human TRPV1 (rTRPV1, hTRPV1) (Liao et al., 2013), rat TRPV3 (rTRPV3), rabbit TRPV2 (rbTRPV2) (Zubcevic et al., 2016) and human TRPA1 (hTRPA1) (Paulsen et al., 2015) were aligned by MUSCLE 3.8. Green boxes mark the selectivity filters. (**B**, **C**) Models for TRPY1 based on xenopus TRPV4 ((Deng et al., 2018), PDB: 6BBJ). For the structural alignment, residues 601-700 in TRPV4 were replaced by residues 367-466 from TRPY1. The aspartate residues 398, 401, 405, 425 and glutamate residues 428 and 429 facing the yeast vacuole lumen are indicated by red sticks, Ca^{2+} by red balls. (**B**) Side view of the S5, pore loop and S6 domains of two opposite TRPY1

wild-type subunits in the absence (left) and presence (right) of Ca^{2+}. (**C**) Top view of the S5, pore loop and S6 domains of four TRPY1 wild-type subunits in the absence and presence of Ca^{2+} (left), and four non-Ca^{2+} binding $TRPY1_{D401A}$ and $TRPY1_{D405A}$ subunits (right). Note, that the putative lower gate, represented by the residues I455 and Y458 (see B), narrows significantly upon Ca^{2+} binding at residues D401 and D405 in TRPY1 wild-type (**B**, **C**).

Transparent Methods

Yeast cell cultures and expression plasmids

For patch clamp and Ca^{2+} Imaging, *Saccharomyces cerevisiae* cells (wild-type strain: W303; *YVC1*-deficient strain: *YVC1::TRP1* in W303, GSY1180) were cultured overnight in liquid YPD or SD media (both Sigma Aldrich, St. Louis, US) at 30°C with rotary shaking. The cells were harvested at an OD_{600} between 1.2 and 1.8. The *YVC1* gene encoding wild-type TRPY1 (NCBI accession number NM_001183506.1) was subcloned into the pRS316 vector (Sikorski and Hieter, 1989) with its own promoter and terminator sequence. All expression plasmids used in this study for transformation in *YVC1*-deficient yeast cells are summarized in Table 1. For Ca^{2+} imaging, these cells were co-transformed with plasmid pEVP11-AEQ89 (Batiza et al., 1996) encoding the Ca^{2+} indicator aequorin.

Antibodies, Western blots and cell surface biotinylation

The rat monoclonal anti-TRPY1 antibody (Chang et al., 2010) was generated in-house using amino acid residues 577 to 675 (C-terminus) of the TRPY1 protein, affinity-purified and used at a dilution of 1:500 for Western blotting. Cell lysates were prepared (Wright et al., 1989), proteins separated by sodium dodecyl sulfate polyacrylamide gel electrophoresis and blotted onto polyvinylidene difluoride (PVDF) membranes (Thermo Fisher Scientific, Schwerte, Germany). Proteins were detected with horseradish peroxidase-coupled secondary antibodies and the Western Lightning Chemiluminescence Reagent Plus (Perkin Elmer) for HEK-293 cells and the SuperSignal West Femto Maximum Sensitivity Substrate (Thermo Fisher Scientific) for yeast. Original scans were saved as TIFF files from LAS 3000 (Fujifilm), which were further processed in Adobe Photoshop. Images were cropped,

resized proportionally, and brought to the resolution required for publication. For yeast TRPY1 wild-type and mutant protein densitometric quantification, backgrounds of the Western blots were subtracted and TRPY1 signal intensities were normalized to the signal intensity of the loading control SRP1. The following additional antibodies were used (dilution, company): Anti-rat peroxidase-conjugated goat antibody (1:2,000, Sigma-Aldrich, Taufkirchen, Germany). anti-aequorin rabbit polyclonal antibody (1:1,000, Abcam, Cambridge, UK), anti-rabbit goat peroxidase-conjugated IgG (1:10,000, Santa Cruz Biotechnology, Dallas, TX, USA), anti-Srp1 rabbit antibody (1:1,000, (Gorlich et al., 1996)). Cell-surface protein biotinylation was essentially performed as described in (Fecher-Trost et al., 2013).

Ca^{2+} imaging in yeast cells

The aequorin photoprotein was used to measure cytosolic free [Ca^{2+}] in yeast cells (Denis and Cyert, 2002). Cells were resuspended in fresh medium to a density of OD_{600}=10. Coelenterazine (Synchem, Felsberg, Germany) was added to a cell suspension of 700 to 1,000 µL at a final concentration of 60 µM. After incubation for 20 min at 30°C, cells were pelleted and resuspended in 700-1,000 µL of fresh medium and incubated again for 45-90 min at 30°C on a roller. 100 µL of the cell suspension were placed into microplate wells and the basal level of luminescence was detected for 30 s at 30°C using a microplate reader (Infinite M200, Tecan, Männedorf, Switzerland). Thereafter 100 µL media containing 3 M NaCl were added yielding a final NaCl concentration of 1.5 M ("hyperosmotic shock"). The luminescence intensity was monitored at 470 nm and plotted as relative luminescence units (RLU) over time using the i-control 1.7 microplate reader software (Tecan). The relative luminescence units obtained for TRPY1 mutants were

normalized to the luminescence units obtained by cells expressing the wild-type *YVC1* cDNA at the same day.

Preparation of giant vacuoles, and whole-vacuole and excised vacuolar patch clamp

Spheroplasts were prepared as described (Bertl and Slayman, 1990). Briefly, yeast cells were incubated in 3 mL of incubation buffer (50 mM KH_2PO_4, 0.2% β-mercaptoethanol, pH 7.2) for 15 min. In order to remove the cell wall 4 mL of protoplasting buffer (50 mM KH_2PO_4, 0.2% β-mercaptoethanol, 2.4 M sorbitol, pH 7.2) including 150 mg bovine serum albumin (fraction V protease-free; Carl Roth, Karlsruhe, Germany) and zymolyase 20T (ICN Biochemicals, Costa Mesa, USA) to a final concentration of 1 mg/mL were added. After 45 min incubation at 30°C on a roller, spheroplasts were harvested and resuspended in stabilizing buffer (220 mM KCl, 10 mM $CaCl_2$, 5 mM $MgCl_2$, 5 mM 2(N-Morpholino)ethanesulfonic acid (MES), 1% (w/v) glucose, pH 7.2). Spheroplasts were incubated for 1 to 2 days to expand and form large vacuoles. The plasma membranes were released by releasing buffer (100 mM potassium citrate, 5 mM $MgCl_2$, 10 mM glucose, 10 mM MES, pH 6.8). After washing with bath solution (see below), the vacuoles were used for whole-vacuole patch clamp experiments.

Break-in was performed by short voltage pulses (850-1,100 mV, 1-5 ms) and currents were recorded using an EPC-9 patch clamp amplifier (HEKA, Lambrecht, Germany). Experiments were performed at an axiovert 135 microscope (Zeiss, Oberkochen, Germany) equipped with a 40x LD Achroplan objective (Zeiss), a 470 nm LED (Rapp OptoElectronics, Hamburg, Germany) and a GFP filter set (Zeiss). Patch pipettes were pulled from glass capillaries GB150T-8P (Science Products, Hofheim, Germany) with a PC-10 micropipette puller (Narishige, Tokyo, Japan) and

after filling with internal solution had resistances between 2 and 4 MΩ. Both internal (patch pipette = vacuolar) and external (bath = cytosolic) saline contained 150 mM KCl, 5 mM $MgCl_2$, 2 mM dithiothreitol (DDT), 10 mM HEPES, pH 7.2. In some experiments, various concentrations of $CaCl_2$, $BaCl_2$, $SrCl_2$, or $MnCl_2$ were added either in the patch pipette, or in an application pipette for direct application onto the measured vacuole. For K^+-free and low Cl^- conditions, KCl was substituted by tetraethylammonium (TEA) chloride or potassium gluconate, respectively.

Voltage ramps of 200 ms spanning from 150 to -150 mV were applied every 2 s from a holding potential (V_h) of 0 mV. From the individual ramp recordings, inward and outward currents were extracted at -80 mV and 80 mV, respectively, and plotted versus time. Representative current-voltage relationships (IVs) were extracted at the stable phase of the current. Currents were normalized to the vacuolar capacitance, which was extracted as a representative measure for the size of the vacuole to calculate current densities (pA/pF). Single channel current amplitudes were measured at -40 mV from current traces of voltage ramps (150 to -150 mV, 200 ms, V_h 0 mV) and voltage steps (-40 mV, 2 s, V_h 0 mV), and at 40 mV from current traces of voltage steps (40 mV, 2 s, V_h 0 mV) in whole-vacuoles and in excised (outside-out) vacuolar patches. Please note that the membrane potentials refer to the cytosolic side, i.e. inward currents at -80 mV represent the movement of positive charges from the vacuole towards the cytosol (Bertl et al., 1992).

Site-directed mutagenesis, modelling and transfection of HEK-293 cells

The *YVC1* cDNA, encoding wild-type TRPY1 (NM_001183506.1) was used as template for site-directed mutagenesis using the Q5 site-directed mutagenesis kit (New England BioLabs, Ipswich, USA). All DNAs were sequenced on both strands.

Table 1 summarizes all expression plasmids used in this study. Figure S6B and C were prepared using PyMOL Molecular Graphics System (Version 1.5.0.4, Schrödinger, LLC, New York, NY, USA) by Coot (Emsley et al., 2010), based on the coordinates of *xenopus* TRPV4 (PDB: 6BBJ; (Deng et al., 2018)). The full-length of *xenopus* TRPV4 tetrameric structure was used as template. Amino acids 601 to 700 of TRPV4 were replaced by amino acids 367 to 466 (S5, pore linker, S6) from yeast TRPY1 (wild-type, D401A and D405A) in Coot. The structural model of wild-type TRPY1 was calculated in the absence and presence of Ca^{2+} in close proximity to D401 and D405.

HEK-293 cells (ATCC, CRL 1573) were cultured in DMEM (Dulbecco's modified eagle medium, Thermo Fisher, Waltham, USA), 10% fetal bovine serum (FBS), 1% penicillin/streptomycin). For patch clamp experiments the HEK-293 cells were transfected with 2 µg of the bicistronic *pCAGGS-IRES-GFP* vector (mock) or plasmids coding for TRPY1 wild-type or the mutants (see Table 1). Fugene HD (Promega, Madison, USA) Lipofectamine 2,000 or 3,000 (Invitrogen, Carlsbad, USA) and 293 Cell Avalanche (EZ Biosystems, College Park, USA) were used as transfection reagents. For whole-cell patch clamp HEK-293 cells were plated on glass cover slips and used for experiments 24-72 h after transfection. The positively transfected HEK-293 cells were identified by their expression of GFP.

Whole-cell patch clamp experiments in HEK-293 cells

Whole-cell currents were recorded using the same system and software as described for yeast vacuolar patch clamp. Voltage ramps of 400 ms spanning from -100 to 100 mV were applied every 2 s from a holding potential of 0 mV. Currents were filtered at 2.9 kHz and digitized at 400 ms intervals. From the individual ramp recordings, inward and outward currents were extracted at -80 mV and 80 mV,

respectively, and plotted versus time. Representative current-voltage relationships (IVs) were extracted at the indicated time points. Currents were normalized to the cell capacitance to calculate current densities (pA/pF). Patch pipettes were filled with internal saline comprising 120 mM Cs-glutamate, 8 mM NaCl, 1 mM $MgCl_2$, 10 mM Cs-BAPTA, 10 mM HEPES (pH 7.2). Ca^{2+} concentrations in the pipette solution were adjusted by the combination of 10 mM Cs-BAPTA with 3.1, 8.2, 9.3, and 9.8 mM $CaCl_2$ to reach final free Ca^{2+} concentrations of 100 nM, 1 µM, 3 µM, and 10 µM, respectively, calculated by WEBMAXC STANDARD (www.stanford.edu). The external solution comprised 140 mM NaCl, 2.8 mM KCl, 2 mM $MgCl_2$, 10 mM HEPES, 10 mM glucose (pH 7.2) with or without 1 or 2 mM $CaCl_2$. Hyperosmotic shock was applied directly onto the measured cells using an application pipette containing 0.5 or 0.75 M sorbitol solved in external solution.

$^{45}Ca^{2+}$ binding

25-mer peptides as indicated were synthesized (Intavis ResPepSL peptide spot synthesizer) and spotted at approximately 16 nmoles per spot) onto hardened cellulose membranes. The membranes were activated by methanol (5 min), washed with distilled water and soaked 120 min in buffer containing 60 mM KCl, 5 mM $MgCl_2$ and 10 mM imidazole-HCl, (pH 6.8), changed every 30 min. Then, membranes were incubated for 30 min in the latter buffer containing additional 1.5 µM (1mCi/L) $^{45}Ca^{2+}$, subsequently rinsed with 50% ethanol for 5 min and dried at room temperature for 3 h. After exposure of the dried membrane to a phosphorimager screen (BAS-IP MS 2040, Fujifilm, Japan) for 12-24 h the screen was scanned by a Typhoon imager (Typhoon FLA 9500, GE Healthcare Life Sciences, USA)

Analysis and Statistics

Patch clamp data were first analyzed in Patchmaster or Fitmaster (HEKA), then transferred to IgorPro (WaveMetrics, Portland, USA) for further analysis and graphical presentation. Yeast luminometric Ca^{2+} imaging data were transferred as Excel files to IgorPro to prepare graphs. IgorPro or GraphPad PRISM (GraphPad, La Jolla, USA) were used to prepare the bar graphs and to test for statistically significant differences between the means of the independent groups with one-way analysis of variance (ANOVA). ns, *, **, *** represents p values of $p > 0.05$, $p \leq 0.05$, $p \leq 0.01$, $p \leq 0.001$, respectively. The error bars represent the standard error of the mean (S.E.M.). Final figures were prepared in CorelDRAW (Corel Corporation, Ottawa, Canada).

Supplemental References

Batiza, A.F., Schulz, T., and Masson, P.H. (1996). Yeast respond to hypotonic shock with a calcium pulse. J Biol Chem *271*, 23357-23362.

Bertl, A., Blumwald, E., Coronado, R., Eisenberg, R., Findlay, G., Gradmann, D., Hille, B., Kohler, K., Kolb, H.A., MacRobbie, E.*, et al.* (1992). Electrical measurements on endomembranes. Science *258*, 873-874.

Bertl, A., and Slayman, C.L. (1990). Cation-selective channels in the vacuolar membrane of Saccharomyces: dependence on calcium, redox state, and voltage. Proc Natl Acad Sci U S A *87*, 7824-7828.

Chang, Y., Schlenstedt, G., Flockerzi, V., and Beck, A. (2010). Properties of the intracellular transient receptor potential (TRP) channel in yeast, Yvc1. FEBS Lett *584*, 2028-2032.

Deng, Z., Paknejad, N., Maksaev, G., Sala-Rabanal, M., Nichols, C.G., Hite, R.K., and Yuan, P. (2018). Cryo-EM and X-ray structures of TRPV4 reveal insight into ion permeation and gating mechanisms. Nat Struct Mol Biol *25*, 252-260.

Denis, V., and Cyert, M.S. (2002). Internal Ca(2+) release in yeast is triggered by hypertonic shock and mediated by a TRP channel homologue. J Cell Biol *156*, 29-34.

Emsley, P., Lohkamp, B., Scott, W.G., and Cowtan, K. (2010). Features and development of Coot. Acta Crystallogr D Biol Crystallogr *66*, 486-501.

Fecher-Trost, C., Wissenbach, U., Beck, A., Schalkowsky, P., Stoerger, C., Doerr, J., Dembek, A., Simon-Thomas, M., Weber, A., Wollenberg, P.*, et al.* (2013). The in vivo TRPV6 protein starts at a non-AUG triplet, decoded as methionine, upstream of canonical initiation at AUG. J Biol Chem *288*, 16629-16644.

Gorlich, D., Kraft, R., Kostka, S., Vogel, F., Hartmann, E., Laskey, R.A., Mattaj, I.W., and Izaurralde, E. (1996). Importin provides a link between nuclear protein import and U snRNA export. Cell *87*, 21-32.

Liao, M., Cao, E., Julius, D., and Cheng, Y. (2013). Structure of the TRPV1 ion channel determined by electron cryo-microscopy. Nature *504*, 107-112.

Paulsen, C.E., Armache, J.P., Gao, Y., Cheng, Y., and Julius, D. (2015). Structure of the TRPA1 ion channel suggests regulatory mechanisms. Nature *520*, 511-517.

Sikorski, R.S., and Hieter, P. (1989). A system of shuttle vectors and yeast host strains designed for efficient manipulation of DNA in Saccharomyces cerevisiae. Genetics *122*, 19-27.

Singh, A.K., Saotome, K., and Sobolevsky, A.I. (2017). Swapping of transmembrane domains in the epithelial calcium channel TRPV6. Sci Rep *7*, 10669.

Wright, A.P., Bruns, M., and Hartley, B.S. (1989). Extraction and rapid inactivation of proteins from Saccharomyces cerevisiae by trichloroacetic acid precipitation. Yeast *5*, 51-53.

Zubcevic, L., Herzik, M.A., Jr., Chung, B.C., Liu, Z., Lander, G.C., and Lee, S.Y. (2016). Cryo-electron microscopy structure of the TRPV2 ion channel. Nat Struct Mol Biol *23*, 180-186.
